# Disparities in HIV Clinical Outcomes among a Cohort of HIV-Infected Persons Receiving Care—Mississippi

**DOI:** 10.3390/ijerph14040392

**Published:** 2017-04-07

**Authors:** Ali Dehghani Firouzabadi, Tiffany C. McDonald, Tametria R. Samms, Reza Sirous, Kendra Johnson

**Affiliations:** 1 Mississippi State Department of Health, Office of STD/HIV, 570 E. Woodrow Wilson, Osborne 200, Jackson, MS 39216, USA; tiffany.mcdonald@msdh.ms.gov (T.C.M.); tametria.samms@msdh.ms.gov (T.R.S.); kendra.johnson@msdh.ms.gov (K.J.); 2 University of Mississippi Medical Center, 2500 N State St, Jackson, MS 39216, USA; rsirous@umc.edu

**Keywords:** the human immunodeficiency virus clinical outcomes, disparities, viral load suppression, durable viral load, cluster of differentiation 4

## Abstract

Increasing patients’ cluster of differentiation 4 (CD4) count and achieving viral suppression are the ultimate goals of the human immunodeficiency virus (HIV) care and treatment, yet disparities in these HIV clinical outcomes exist among subpopulations of HIV-infected persons. We aimed to assess potential disparities in viral suppression and normal CD4 count among HIV-infected persons receiving care in Mississippi using Mississippi Medical Monitoring Project (MMP) data from 2009 to 2014 (*N* = 1233) in this study. Outcome variables in this study were suppressed, recent and durable viral load, and normal CD4 count. Patients’ characteristics in this study were race, gender, age, annual income, education, insurance, and length of diagnosis. Descriptive statistics, Chi square tests, and logistic regression analyses were conducted using the SAS 9.4 Proc Survey procedure. Our findings indicate that those aged 50 years or older were more likely to have suppressed recent viral load (adjusted Odds Ratio (aOR) = 2.4) and durable viral loads (aOR = 2.9), compared to those aged 18–24 years. In addition, women were more likely to have a normal CD4 count than men (aOR = 1.4). In conclusion, we found that age and gender disparities in HIV clinical outcomes may be used to develop and implement multifaceted interventions to improve health equity among all HIV-infected patients.

## 1. Introduction

Over the past few decades, multiple strategies have been implemented worldwide to optimize the human immunodeficiency virus (HIV) care, and improve the quality of life among people living with HIV/acquired immune deficiency syndrome (AIDS). Antiretroviral medications, arguably one of the most efficacious innovations of recent years, have brought a remarkable reduction in HIV-related mortality and morbidity in both adults and children living with HIV/AIDS, and have transformed HIV infection into a manageable chronic disease [1]. Two key goals of the HIV care continuum are achieving viral suppression and increasing cluster of differentiation 4 (CD4) counts. CD4 counts and HIV viral load are the best indicators for monitoring the immune system and clinical progression of HIV. Previous studies indicate that increase in CD4 count and suppression of viral load to undetectable levels have several beneficial health outcomes, including enhanced physical functioning, reduced infections, improved quality of life, and reduced HIV-related mortality [2,3].

Despite these care and treatment advancements, southern states in the U.S., including Mississippi, have been disproportionately affected by the HIV endemic in terms of the overall number of people living with HIV/AIDS, as well as survival rates after HIV/AIDS diagnosis, and lag far behind in providing quality HIV/AIDS care and prevention. Southern states account for nearly half of all people living with an HIV diagnosis in the U.S. (44%), despite making up only about one-third (37%) of the overall U.S. population [4]. Mississippi ranked ninth in the nation for diagnosed HIV infection in 2014; however, the number of new diagnoses remain fairly stable in recent years, with a 5% increase in cases from 2010 to 2014 (average of 482 cases each year) [5].

Disparities in HIV health outcomes have been studied in some states [6,7]; however, there is limited information about disparities in HIV health outcomes and their associated factors in Mississippi. To develop tailored and multifaceted strategies for effective intervention, more detailed information regarding the characteristics and health outcomes of HIV-infected individuals in Mississippi is needed. Addressing this information gap, we sought to assess potential disparities in two major indicators of HIV care outcomes, suppressed viral load and normal CD4 count, and the explanatory factors therein among HIV-infected persons receiving care in Mississippi during the period 2009–2014.

## 2. Methods

### 2.1. Study Design and Participants

We used Mississippi Medical Monitoring Project (MMP) data from 2009 to 2014 (*n* = 1233) to assess viral load suppression and to identify potential disparities in viral load among a cohort of HIV-infected persons in care in Mississippi. MMP is an on-going, nationally representative, cross-sectional surveillance system designed to assess and monitor the behavioral and clinical characteristics of HIV-infected adults at least 18 years of age receiving outpatient medical care for HIV in the United States and Puerto Rico. MMP is funded by the Centers for Disease Control and Prevention (CDC) and is conducted across the nation in collaboration with the National Institutes of Health (NIH), the Health Resources and Services Administration (HRSA), and state and local health departments. The main data collection procedures include in-person interviews of eligible patients, and abstraction of their HIV-related medical records. Interview data provide information on patients’ demographics, access and barriers to HIV-related secondary prevention services, met and unmet needs of HIV-related medical services and utilization of these services, and patients’ current behaviors that may facilitate HIV transmission such as sexual behaviors and injection drug use. In combination with data collected from the abstraction of HIV-related medical records, the MMP provides information on clinical conditions that result from HIV-infected persons’ disease or the medications they take, as well as the type and quality of HIV care and support services being received by these patients [8].

To select a representative sample of approximately 400 persons receiving primary HIV medical care, MMP utilizes the three-stage probability proportional-to-size sampling process: (1) project areas; (2) facilities providing outpatient HIV medical care in selected project areas including any clinic, health care facility, and group or private physician practice; and (3) HIV-infected persons receiving medical care at selected facilities. Providing HIV care, an eligibility requirement for the facility sampling frame, is operationally defined as conducting CD4 or HIV viral load testing and/or providing prescriptions for antiretroviral medications, HIV counseling or any other medical care for treatment and management of HIV. The facilities that are known not to provide medical care; those that obtain CD4 counts and HIV viral loads only for referral purposes; or those that only provide antiretroviral refill prescriptions but do not have an active role in HIV management are excluded from MMP sampling. To be eligible for MMP, (1) patients must be diagnosed with HIV, (2) be at least 18 years of age, (3) have received medical care (defined as any visit to a facility that provides HIV care for medical care or prescription of medications, including refill authorizations) at the sampled facility, and (4) able to provide informed consent [9]. All HIV-infected adults who participated in the 2009 to 2014 cycles of Mississippi MMP were eligible for inclusion, and informed consent was obtained from all participants. The 2009–2014 Mississippi MMP questionnaires were approved by the Institutional Review Boards of the CDC and the Mississippi State Department of Health.

### 2.2. Statistical Analyses

To account for the stratified and weighted sample, we analyzed data using the survey procedures in SAS 9.3 (SAS Institute, Inc., Cary, NC, USA). We examined three outcome variables: (1) suppressed recent viral load; (2) suppressed durable viral load, which was all defined viral load results over the past 12 months documented in the medical record as undetectable; and (3) Normal CD4 count. Viral load is a measure of how much HIV virus is in a person’s blood, and undetectable viral load in this study was defined as viral load <200 copies per milliliter. CD4 count, a strong indicator of immune system function, is the number of CD4 T-lymphocytes in a blood sample. In this study, the mean normal value for CD4 count was ≥500 cells/mm^3^. Descriptive statistics were used to summarize baseline sample characteristics by viral load suppression and normal CD4 counts. Patients’ characteristics in this study were race (white and black), gender (male and female), age (18–29, 30–39, 40–49, ≥50 years), annual income (<USD $20,000 and ≥$20,000), education (<High School and ≥High School), insurance (private, public, uninsured), and length of diagnosis (<5 years and ≥5 years). The distribution of HIV-infected persons receiving medical care characteristics was summarized using unweighted counts and weighted percentages. All proportions represent weighted percentages that are generalized to all HIV-infected persons residing in Mississippi and receiving outpatient medical care between January to April of 2009–2014. Multiple logistic regression models were developed to provide an estimate of associations between outcome variables and patient characteristics. Although the odds ratio is prone to overestimating the magnitude of the association between the outcome and exposure when the outcome of interest is common (>20%) [10], for retrospective studies with binary outcomes that require adjustment for covariates and interaction estimations, the odds ratio is preferred because it has desirable mathematical properties and will not be misleading when interpreted correctly [11]. For the unadjusted model, we measured the unadjusted effects of each category of patients’ characteristics and the likelihood of suppressed viral load and normal CD4 count. In the adjusted model, we measured the independent effects of each category after controlling for all confounders. Variables were included in the multiple logistic regression model if their *p*-value was less than 0.20 on bivariate analysis. All analyses accounted for the complex sample design and unequal selection probabilities.

## 3. Results

Demographics of the study sample are shown in Table 1. The majority of respondents were male (63.5%), black (82.2%), 40–49 years (32.7%), had high school education or more (73.9%), had annual income less than $20,000 (86.2%), had public insurance (53.9%), and were diagnosed with HIV 5 years ago or more (71.5%). Nearly 65% had an undetectable recent viral load, and 58.1% had an undetectable durable viral load. Less than half of the participants had CD4 counts ≥500 cells/mm^3^ (41.5%).

Patients’ characteristics by viral load suppression and CD4 counts ≥500 cells/mm^3^ are listed in Table 2. According to this table, recently suppressed viral load prevalence was significantly higher among whites, those aged 50 years or older, those who had annual income $20,000 or more, had public insurance, and those who were diagnosed at least 5 years ago. Those 50 years of age or older and those who were diagnosed 5 years ago or more had the highest proportion of having durable viral load suppression. Gender was the only variable significantly associated with CD4 counts ≥500 cells/mm^3^. Women had higher prevalence of CD4 counts ≥500 cells/mm^3^ compared to men.

In the multivariable analysis reported as adjusted Odd Ratio (aOR) (Table 3), persons more likely to have recently suppressed viral loads were 50 years of age or older, compared with those aged 18–24 years (aOR = 2.4, 95% Confidence Interval (CI): 1.5–3.7). Although white HIV-infected persons were more likely to report suppressed recent viral load, the association between race and recent viral suppression was not significant. Similarly, age was significantly associated with durable viral load suppression. Those aged 50 years or older were more likely to have durable viral load suppression compared to those aged 18–24 years (aOR = 2.9, 95% CI: 1.7–5.1). For CD4 counts ≥500 cells/mm^3^, women were more likely to have CD4 counts ≥500 cells/mm^3^ compared to men (aOR = 1.4, 95% CI: 1.1–1.7).

## 4. Discussion

This study is the first to examine the disparities in viral load suppression and CD4 counts among HIV-infected adults in care in Mississippi. Using the MMP standardized sampling methodology and data collection instrument, we found disproportionate viral load suppression and normal CD4 count among people receiving care for HIV/AIDS in this state. Overall, the majority of participants had suppressed recent and durable viral load. The prevalence of suppressed recent viral load was significantly higher among whites, those 50 years of age or older, those with an annual income $20,000 or more, those with public insurance, or those who were diagnosed at least 5 years ago. On the other hand, those who were 50 years of age or older and were diagnosed 5 years ago or more had significantly the highest prevalence of durable viral load suppression. Also, less than half of the participants had higher immune status, as indicated by a normal CD4 count (≥500 cells/mm^3^). Women had a significantly higher prevalence of normal CD4 count compared to men.

Similar to previous studies [12,13], we found that there were age disparities in viral load suppression. Our findings indicate that older patients were more likely to have suppressed recent and durable viral loads. One possible reason for these age disparities could be delayed screening and diagnosis among young adults, resulting in higher viral load at baseline of diagnosis compared to older adults. Previous studies show that nearly half of young adults delay care until their disease has advanced [14]. Some research suggests that young adults experience more HIV-related stigma, fear of disclosure, and stress than older adults, increasing delays in being diagnosed and accessing care [15]. In addition, younger HIV-infected patients are more likely to have lower antiretroviral therapy adherence compared to older patients due to lack of disease management knowledge and skills, lack of perceived treatment benefits, and lower self-efficacy, resulting in higher HIV viral load [16]. Younger age is generally associated with less comorbidities, and consequently less experience with taking medications leading to higher perception of treatment difficulty, lower disease management and self-efficacy in younger adults than their older counterparts. In addition, taking medication may require more changes in younger adults’ lifestyle due to their busy and varying schedules and make regular adherence more challenging as they may be less able to integrate medications into their daily routine [17]. Regimen simplification may improve their quality of life and increase youth perceived self-efficacy in managing their medication, and help them maintain long-term adherence. Lower Antiretroviral therapy (ART) adherence in younger adults with HIV may be due to their more frequent substance abuse when compared to older adults. Neurocognitive impairment of drug abusers and their unpredictable, and chaotic lifestyle make may limit their attention to timing of doses or to dietary consideration leading to poor adherence [18,19]. To address this issue, it may be helpful to provide simple daily reminders as well as increase their access to treatment for substance abuse.

We also explored potential disparities in HIV-infected patients’ immune status and found gender disparities in having a normal CD4 count. Our observations that women had a higher proportion of normal CD4 than men are consistent with findings from previous studies. These studies suggest that gender disparities in normal CD4 count may be attributed to late diagnosis and care initiation. Compared to women, men are at higher risk of delaying care at more advanced disease stages and have poorer outcomes even after ART initiation [20,21]. Many studies have reported greater use of primary healthcare services in women [22,23]; therefore, women are more likely to utilize counseling and testing services as part of routine health care, and also more likely to be offered HIV testing by healthcare providers due to prenatal HIV screening, family planning, as well as gynecological follow-up [24]. In addition, women have higher risk perception of HIV compared to men, which might influence them to get tested and diagnosed earlier in their diagnosis [25].

Although the associations between race and HIV clinical outcomes were not statistically significant, our findings show that whites were more likely to have suppressed recent and durable viral load, and a normal CD4 count. Similarly, those who had $20,000 annual income or more were more likely to have suppressed recent and durable viral load; however, the associations between viral load suppression and income were not statistically significant. Moreover, HIV-infected persons who were diagnosed with HIV infection at least 5 years ago were more likely to have suppressed durable viral load. These non-significant results may be due to the small sample size and need to be further investigated. However, from a clinical perspective, non-significant outcomes may provide valuable information and should not be ignored. These non-significant outcomes may help health professionals better understand and utilize the evidence to make a better care decision and to deliver better services [26].

This research presents new insight into the condition of HIV clinical outcomes and associated disparities in Mississippi. The results provide public health professionals with valuable information about HIV-infected individuals who are at higher risk for poor outcomes and highlight a need for eliminating disparities to promote healthy outcomes among patients with HIV. A particular strength of this study is the use of a population-based sample that is weighted to reflect the total HIV-infected people receiving care in Mississippi. However, our analysis is subject to several limitations. First, the study’s population was limited to those receiving HIV medical care; therefore, it cannot be generalized to all HIV-infected persons in Mississippi. To address this limitation, MMP started collecting data from all people living with HIV regardless of their care status in 2015. The expansion of MMP to include HIV-infected persons not in care may lead to better understanding of factors that affect care utilization and the development of effective interventions for improving access to care for all people with HIV. Second, although the study population was limited to those who are receiving medical care, some participants may not currently take antiretroviral medications or could have had periods of antiretroviral medications discontinuation while still in care. Lack of information on sustained antiretroviral therapy utilization and discontinuation of antiretroviral medications in this analysis could be another limitation of this study. Third, MMP is a cross-sectional study, thus causality and the direction of results cannot always be determined. Fourth, part of the data of this analysis were obtained from surveys and are self-reported and might be subject to recall and social desirability bias. In addition, data abstracted from medical records may be prone to recording errors. Finally, although MMP collects many behavioral and clinical information, our logistic regression models considered only a limited number of factors because of the relatively small sample size.

## 5. Conclusions

In conclusion, we found that most HIV-infected persons in medical care in Mississippi were virally suppressed and had a normal CD4 count. Our findings identified differences between groups in these two major indicators of HIV clinical outcomes, which may be used to develop and implement multifaceted interventions to improve health equity among these groups, and address social determinants in order to eliminate health disparities among all HIV-infected patients. Although further studies are needed to fully explain disparities in viral suppression and CD4 among HIV-infected persons, our results suggest early linkage and retention programs, youth-focused delivery of care, and programs that assist adherence may help reduce disparities among HIV-infected persons.

## Figures and Tables

**Table 1:** Characteristics of HIV-infected people in care in Mississippi (*N* = 1233).

Characteristics	n ^a^ (% ^b^)
*Gender*	
Male	739 (63.5)
Female	476 (36.5)
*Race*	
White	203 (17.8)
Black	1003 (82.2)
*Education*	
<High School	331 (26.1)
≥High school	901 (73.9)
*Age (years)*	
18–29	194 (16.3)
30–39	274 (22.2)
40–49	401 (32.7)
≥50	364 (28.9)
*Annual Income*	
<$20,000	976 (86.2)
≥$20,000	155 (13.8)
*Insurance*	
Private	144 (12.5)
Public	671 (53.9)
Uninsured	410 (33.7)
*Time Since HIV Diagnosis*	
<5 years	346 (28.5)
≥5 years	886 (71.5)
*Recent Viral Load*	
Undetectable (<200 copies/mL)	798 (64.9)
Detectable (≥200 copies/mL)	435 (35.1)
*All Viral Load*	
Undetectable (<200 copies/mL)	640 (58.1)
Detectable (≥200 copies/mL)	463 (41.9)
CD4 Count	
No (<500 cells/mm^3^)	697 (58.5)
Yes (≥500 cells/mm^3^)	478 (41.5)

HIV: Human Immunodeficiency Virus; ^a^ Unweighted count; ^b^ Weighted percentage.

**Table 2:** Patients’ characteristics by viral suppression and CD4 counts ≥500 cells/mm^3^.

Characteristics	Suppressed Recent Viral Load	*p*-Value	Suppressed Durable Viral Load	*p*-Value	CD4 Counts ≥500 cells/mm^3^	*p*-Value *
	N ^a^ (% ^b^)		N ^a^ (% ^b^)			
*Gender*		0.96		0.20		0.01
Male	476 (64.8)		373 (56.6)		262 (38.8)	
Female	310 (64.7)		257 (60.4)		207 (46.0)	
*Race*		0.01		0.13		0.16
White	146 (72.8)		114 (62.7)		90 (46.0)	
Black	633 (62.9)		509 (56.6)		375 (40.2)	
*Education*		0.29		0.65		0.80
<High School	208 (62.6)		174 (59.4)		128 (42.1)	
≥High school	590 (65.7)		466 (57.7)		349 (41.3)	
*Age (years)*		0.0002		<0.0001		0.56
18–29	103 (53.7)		78 (45.9)		69 (39.0)	
30–39	169 (61.4)		136 (56.1)		108 (41.4)	
40–49	256 (64.4)		196 (53.7)		147 (40.1)	
≥50	270 (74.3)		230 (71.7)		154 (44.6)	
*Annual Income*		0.02		0.14		0.36
<$20,000	616 (63.4)		499 (57.2)		375 (41.2)	
≥$20,000	118 (76.0)		92 (65.6)		67 (45.7)	
*Insurance*		0.01		0.06		0.70
Private	93 (64.7)		71 (57.9)		57 (42.3)	
Public	457 (68.3)		374 (61.0)		252 (40.3)	
Uninsured	243 (59.3)		190 (52.9)		166 (43.2)	
*Time Since HIV Diagnosis*		0.02		0.003		0.07
<5 years	202 (58.4)		151 (49.4)		119 (37.4)	
≥5 years	595 (67.4)		488 (61.4)		358 (43.1)	

CD4: cluster of differentiation 4; ^a^ Unweighted count; ^b^ Weighted percentage; * *p*-values obtained through Pearson chi-squared.

**Table 3:** Association between patients’ characteristics and HIV clinical outcomes.

Characteristics	Suppressed Recent Viral Load	*p*-Value	Suppressed Durable Viral Load	*p*-Value	CD4 Counts ≥500 cells/mm^3^	*p*-Value
	aOR		aOR			
*Gender*						
Male	-	-	-	-	Reference	
Female	-	-	-	-	1.4 (1.1–1.7)	0.007
*Race*						
White	1.4 (0.98–2.1)	0.05	1.3 (0.89–1.9)	0.17	1.3 (0.94–1.9)	0.11
Black	Reference		Reference		Reference	
*Education*						
<High School	-	-	-	-	-	-
≥High school	-	-	-	-	-	-
*Age (years)*						
18–29	Reference		Reference			
30–39	1.4 (0.94–2.2)	0.08	1.4 (0.89–2.2)	0.14	-	-
40–49	1.5 (0.92–2.4)	0.10	1.2 (0.68–2.1)	0.52	-	-
≥50	2.4 (1.5–3.7)	<0.0001	2.9 (1.7–5.1)	0.0001	-	-
*Annual Income*						
<$20,000	1.8 (0.98–3.2)	0.06	1.3 (0.77–2.3)	0.30	-	-
≥$20,000	Reference		Reference			
*Insurance*						
Private	1.2 (0.73–1.8)	0.53	1.2 (0.76–1.8)	0.44	-	-
Public	1.3 (0.95–1.7)	0.11	1.1 (0.75–1.6)	0.63	-	-
Uninsured	Reference		Reference		Reference	
*Time Since HIV Diagnosis*						
<5 years	Reference		Reference		Reference	
≥5 years	1.1 (0.81–1.5)	0.52	1.4 (0.97–1.9)	0.07	1.2 (0.94–1.6)	0.11

aOR: adjusted Odds Ratio; Note: Variables with *p*-value > 0.20 in bivariate analysis were excluded from the final model.
